# Discovering CRIP1: a novel core gene in osteoarthritis pathogenesis

**DOI:** 10.1186/s41065-025-00576-4

**Published:** 2025-10-17

**Authors:** Qifan Chen, Mengliang Luo, Wenhao Kuang, Xianfang Guo, Hao Wu, Shiqi Wu, Sanmao Liu, Yueliang Wen, Chushong Zhou, Maolin He

**Affiliations:** 1 Department of Spinal Surgery, The First Affiliated Hospital of Guangxi Medical University, Shuangyong Road 6, Nanning, 530021 Guangxi Zhuang Autonomous Region China; 2 Department of Orthopedic, Center for Joint Surgery, The Second Affiliated Hospital of Chongqing Medical University, Yuzhong District, Chongqing, 400010 China; 3 Department of Spinal Surgery, Zhujiang Hospital, Southern Medical University, Guangzhou, 510280 China; 4 Department of Neurosurgery, The Second Affiliated Hospital of Guangxi Medical University, Nanning, 530005 Guangxi Zhuang Autonomous Region China

**Keywords:** Osteoarthritis (OA), Core gene, Diagnostic model, Biomarker, CRIP1

## Abstract

**Background:**

Osteoarthritis (OA) is a prevalent chronic degenerative joint disease characterized by complex pathological mechanisms. This study aims to investigate core genes and their associated pathways in OA cartilage.

**Methods:**

We integrated multiple transcriptome datasets, comprising four microarray datasets and two high-throughput datasets. Key pathways related to OA were identified through differential gene analysis, Gene Ontology (GO) enrichment analysis, Kyoto Encyclopedia of Genes and Genomes (KEGG) enrichment analysis, and Gene Set Enrichment Analysis (GSEA). Subsequently, immune infiltration analysis was conducted to explore infiltration characteristics in cartilage tissue, and 113 machine learning algorithms were utilized to identify core genes. The expression of these genes was subsequently verified by qRT-PCR, and an OA diagnostic model was constructed.

**Results:**

GSEA analysis demonstrated significant activation of the ECM-receptor interaction pathway in OA. Utilizing machine learning analysis, we identified APOD, CRIP1, and S100A4 as core genes, with APOD significantly down-regulated and CRIP1 and S100A4 significantly up-regulated. The diagnostic model based on these three genes exhibited robust predictive ability and clinical applicability.

**Conclusions:**

This study highlights the critical role of the ECM-receptor interaction pathway in OA development and identifies APOD, CRIP1, and S100A4 as key regulatory factors. Notably, the potential role of CRIP1 warrants further investigation, providing a novel direction and theoretical foundation for future OA research.

## Introduction

Osteoarthritis (OA) is a chronic degenerative joint disease, which is characterized by synovial inflammation and hyperplasia, degeneration of articular cartilage, and osteophyte formation. The clinical manifestations of OA include joint pain, swelling and stiffness, and limited movement [1–3]. According to the latest research analysis of the Global Burden of Disease database, the incidence and prevalence of OA will continue to increase, and the cumulative number of cases is expected to reach 38,800,395 by 2040 [4]. At the same time another in musculoskeletal disease prediction research also affirmation of the burden of osteoarthritis showed a trend of rising in the global scope, and the burden of national and regional difference [5]. In 2020, 7.6% of the global population had OA, and this proportion will only increase by 2050 [6].

Chondrocytes play a crucial role in the pathogenesis of osteoarthritis [7, 8]. The aging of chondrocytes not only cannot effectively produce and maintain cartilage matrix, but also accelerate the development of osteoarthritis by affecting mitochondrial function and intracellular environment [9, 10]. There are many forms of chondrocyte death, including apoptosis, pyroptosis, autophagy, ferroptosis and cup-death. No matter what form of chondrocyte death, these cell death pathways not only reduce the number of chondrocytes, but also aggravate joint damage by inducing inflammation and other harmful processes [11–13].

In recent years, by integrating the transcriptome learn and multiple sets of data combined with machine learning techniques, the researchers can screen play a key role in the development of disease genes and pathways, and is verified by experiments in vivo and in vitro [14–16]. These emerging technologies reveal the complex responses of chondrocytes in the process of OA, including gene expression changes and metabolic abnormalities. However, there are some limitations in previous studies, such as the small number of machine learning algorithms, the limited sample size of datasets analyzed, and the relatively simple types of datasets used [17]. These factors lead to significant differences in the core genes screened from different datasets, which limits the generalizations and explanatory power of the results.

This study aims to integrate transcriptome datasets from OA cartilage tissue, including first generation and next generation sequencing technologies, to screen core genes and functional pathways, and to validate the expression changes of these core genes by PCR experiments. In addition, we also constructed a diagnostic model for OA. The strengths of this study include the use of richer data sets (both microarray data and high-throughput data), the increase of sample size (more than one hundred), and the application of more comprehensive machine learning algorithms (113 in total). These results will provide new insights into gene expression and its molecular mechanisms in OA chondrocytes.

## Materials and methods

### Data download and processing

**Fig. 1:**
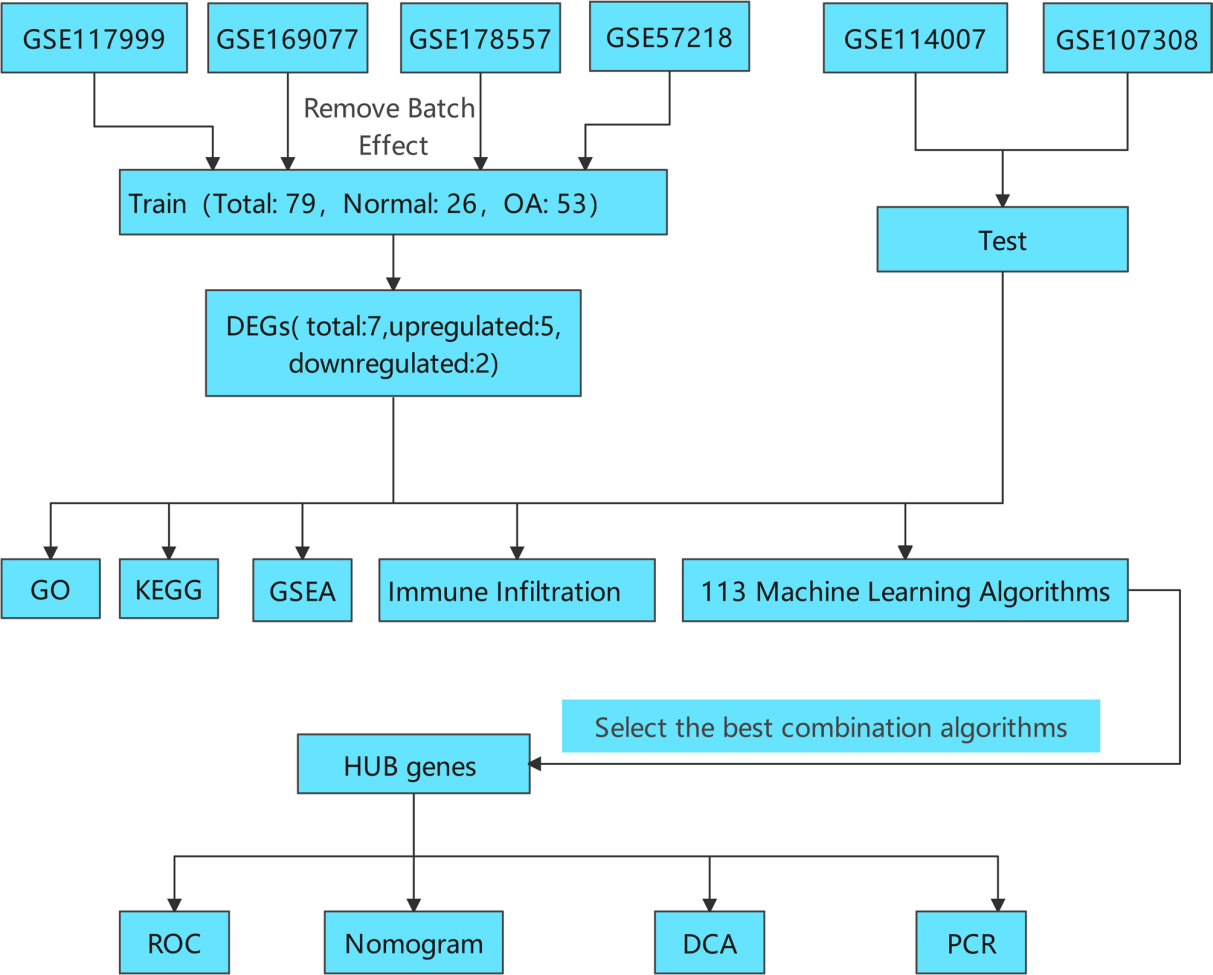
Flowchart of the comprehensive analysis process for the transcriptome of osteoarthritis cartilage

The flow chart of this study is illustrated in Fig. 1. We searched the GEO database (https://www.ncbi.nlm.nih.gov/geo/) using the keywords “osteoarthritis” and “cartilage” to retrieve relevant datasets. A total of six datasets related to osteoarthritis cartilage tissue were downloaded, comprising four microarray datasets (GSE117999, GSE169077, GSE178557, GSE57218) and two high-throughput datasets (GSE114007, GSE107308). Detailed information regarding each dataset is presented in Table 1. We utilized the four microarray datasets as training sets and the two high-throughput datasets as validation sets. To eliminate batch effects, the “sva” package was employed for batch correction of the microarray datasets. The final training set consisted of 79 samples, which included 26 normal samples and 53 osteoarthritis samples.

**Table 1:** Sample information of the OA-Related datasets

Dataset	Platform ID	Normal cartilage	Osteoarthritis cartilage	Sequencing technology	Species
GSE178557	GPL13497	4	4	Microarray	Human
GSE169077	GPL96	5	6	Microarray	Human
GSE117999	GPL20844	10	10	Microarray	Human
GSE57218	GPL6947	7	33	Microarray	Human
GSE107308	GPL16791	6	10	High throughput	Human
GSE114007	GPL11154	18	20	High throughput	Human

### Differential gene analysis

The microarray dataset was processed with the “limma” package, and the high-throughput dataset was processed with the DESeq2 package for differentially expressed gene (DEG) analysis, so as to obtain the differentially expressed genes between normal samples and osteoarthritis samples. The screening criteria were a corrected p-value < 0.05 and |log fold change (FC)|≥1. Subsequently, the “ggplot2” and “pheatmap” packages were used to draw the volcano map and heatmap, respectively, to show the up-regulated and down-regulated differentially expressed genes.

### GO, KEGG enrichment analysis and GSEA analysis

The R package “clusterProfiler” was used for Gene ontology (GO) Analysis, Kyoto Encyclopedia of Genes and Genomes (KEGG) pathway analysis, and Gene Set Enrichment Analysis on the training set and validation set. The screening criteria were all corrected p-value < 0.05.

### Immune cell infiltration analysis

The “cibersort” package was used to analyze the infiltration of immune cells in the cartilage tissue of the training set and the validation set.

The bar chart shows the proportion of different immune cells in OA and normal samples, and the violin plot visually shows the difference of immune cell infiltration between OA patients and normal people.

### Core gene screening and machine learning analysis

According to the differentially expressed genes obtained from the training set, 113 machine learning combination algorithms were further used to analyze the training set and validation set to screen the most important core genes. These 113 algorithms are arranged and combined from 12 basic algorithms. The 12 basic algorithms include LASSO, support vector machine (SVM), Random forest (RF), glmBoost, partial least squares regression (plsRglm), Stepglm, elastic net (Enet), Linear discriminant analysis (LDA), gradient boosting machine (GBM), Ridge regression (Ridge), XGBoos t and Naive Bayes, respectively. Each of these basic algorithms has its own strengths and can adapt to different data characteristics and distributions to improve the overall performance of the model. The R packages used include openxlsx, seqinr, plyr, randomForestSRC, glmnet, plsRglm, gbm, caret, mboost, e1071, BART, MASS, snowfall, xgboost, ComplexHeat map, RColorBrewer, and pROC.

To achieve the best performance, we rely on hyperparameter tuning programs such as grid search and random search to systematically adjust the hyperparameters of each combination; The ensemble learning strategy was used to weighted average the prediction results of each algorithm to improve the robustness of the model and reduce the risk of overfitting. Finally, k-fold cross-validation was used to evaluate the effectiveness of the combination of algorithms to ensure that the model maintained good performance on different data subsets and had strong generalization ability. We calculated the area under the receiver operating characteristic curve (AUC) of the training set and the validation set. The heat map was used to visualize the model results to screen the best performing combination algorithm, and the Receiver operating characteristic (ROC) curve and gene expression of the core genes were plotted.

### Cell culture

Primary chondrocytes were obtained from undamaged areas of the knee joints of patients undergoing total knee arthroplasty. In short, cut the cartilage tissue into fragments (0.5mm^3^). The chopped cartilage pieces were incubated with 0.2% type II collagenase for 18–24 h in a constant temperature shaker at 37 °C. Primary chondrocytes were obtained by filtering through a 70 μm filter and then centrifuging. Chondrocytes were cultured in a T75 cell culture flask with DMEM/F12 medium containing 10% FBS and 1% penicillin-streptomycin. IL-1β was used to interfere with chondrocytes for 48 h to obtain the OA group.

### qR-PCR was used to verify the expression of core genes

Total RNA was isolated from samples using TRIZOL reagent (Invitrogen) according to the manufacturer’s protocol. Then, using the Evo M-MLV RT Kit (AG, China), 1000 ng of total RNA was reverse transcribed into cDNA. qRT-PCR analysis was performed using Power SYBR Green Master Mix (AG, China) according to the manufacturer’s standard protocol. Relative mRNA expression levels were calculated using the 2-ΔΔCt method, with GAPDH serving as the internal reference. All samples were assayed in quintuplicate. The primer sequences utilized in this study are summarized in Table 2.

**Table 2:** Quantitative reverse-transcription PCR primer sequences

Genes	Forward (5’−3’)	Reverse (5’−3’)
GAPDH	GCAAATTCCATGGCACCGT	TCGCCCCACTTGATTTTGG
APOD	TGAAGCCACCCCAGTTAACC	CATAGTCGGTGGCCAGGATC
CRIP1	TCCCAAGTGCAACAAGGAGG	GGGTGGTTGCAGTAGGGTTT
S100A4	CCCTGGATGTGATGGTGTCC	CCTGTTGCTGTCCAAGTTGC

### Construction of diagnostic model

Core genes were utilized to construct reliable diagnostic models. Firstly, a nomogram (NomogsJIAm) and a calibration curve (CalibsJIAtion Curve) were generated using the ‘rms’ package to intuitively illustrate the consistency between the model’s predicted probabilities and the actual outcomes. Furthermore, clinical decision curve analysis (DCA) was performed to assess the net benefits of the model in clinical practice.

### Statistical analysis

All statistical analyses were performed using R version 4.4.1 software and its corresponding packages. A t-test was used to compare data between the two groups, with *p* < 0.05 considered statistically significant.

## Results

### Seven differentially expressed genes were identified

**Fig. 2:**
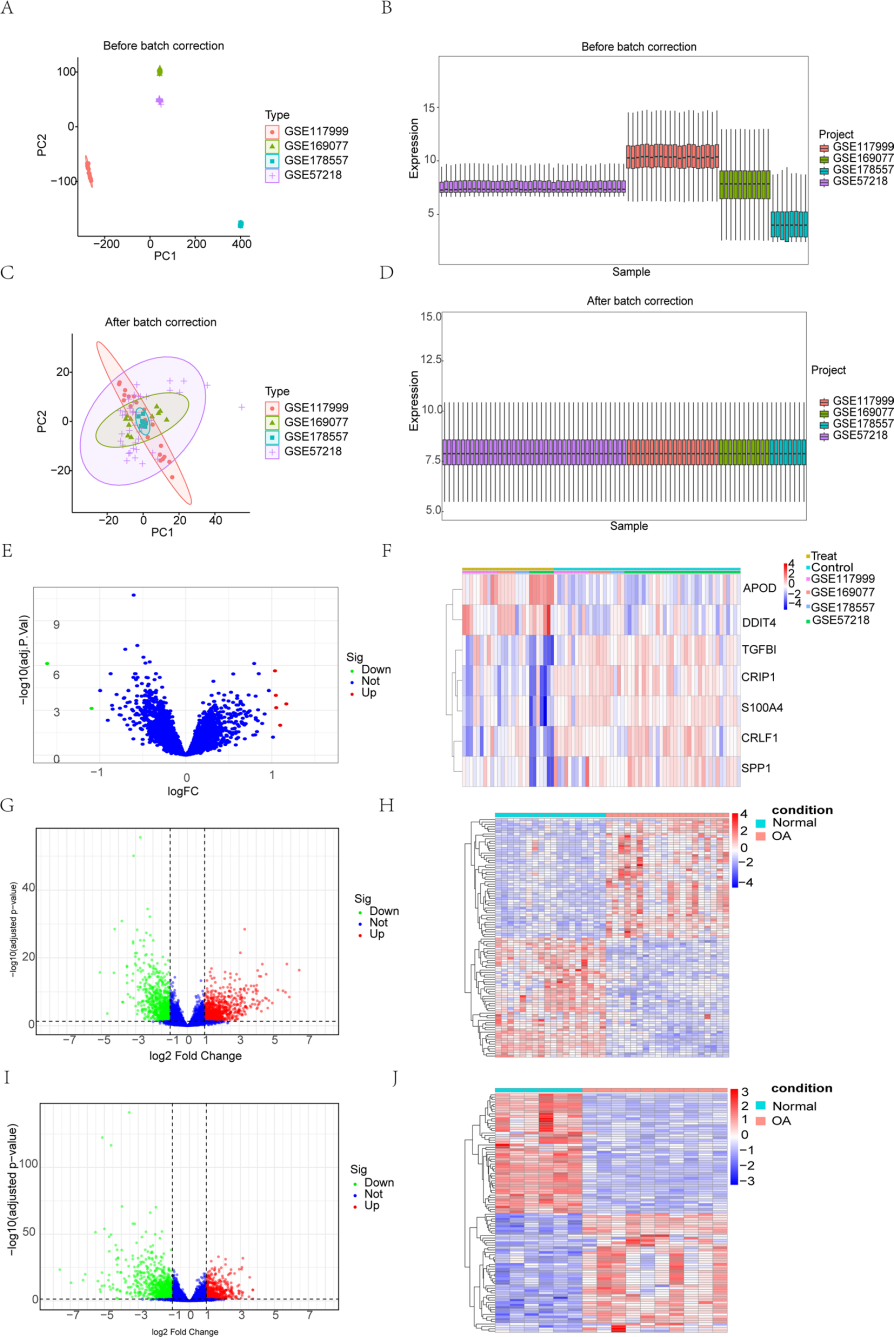
(**A-B**) PCA plot and box plot before batch effect removal; (**C-D**) PCA plot and box plot after batch effect removal; (**E-F**) Volcano plot and heatmap for the training set; (**G-H**) Volcano plot and heatmap for GSE11407; (**I-J**) Volcano plot and heatmap for GSE107308

Before batch effect were removed, the principal component analysis and boxplot show the sample distribution, there is an obvious effect of batch (Fig. 2A, B). The comparability of the data was significantly improved after correcting for batch effects (Fig. 2C, D). A total of seven differentially expressed genes (DEGs) were identified in the training set, with five genes upregulated and two genes downregulated in the volcano plot (Fig. 2E). The heatmap shows the overall characteristics of the DEGs (Fig. 2F). Additionally, the volcano plots and heatmaps of the two high-throughput datasets also display the specific characteristics of their respective differentially expressed genes (Fig. 2G-J).

### Activated pathways were shown by GO, KEGG enrichment analysis and GSEA analysis

**Fig. 3:**
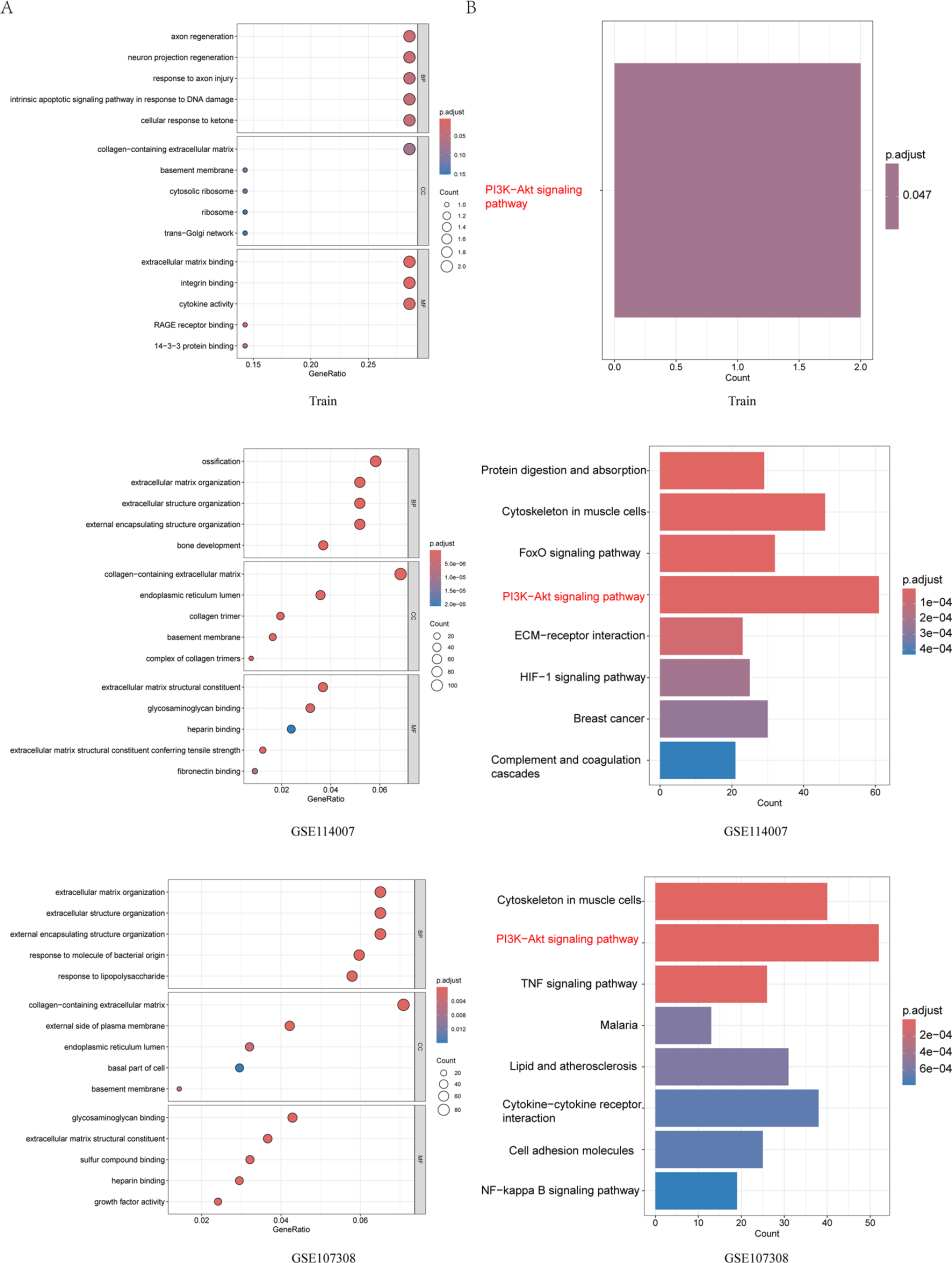
(**A**) GO analysis bubble plot for the training and validation sets, including biological processes, cellular components, and molecular functions. (**B**) Bar chart of KEGG analysis results for the training and validation sets, highlighting that the PI3K-Akt signaling pathway is significantly enriched in all datasets, emphasized in red text in the figure

**Fig. 4:**
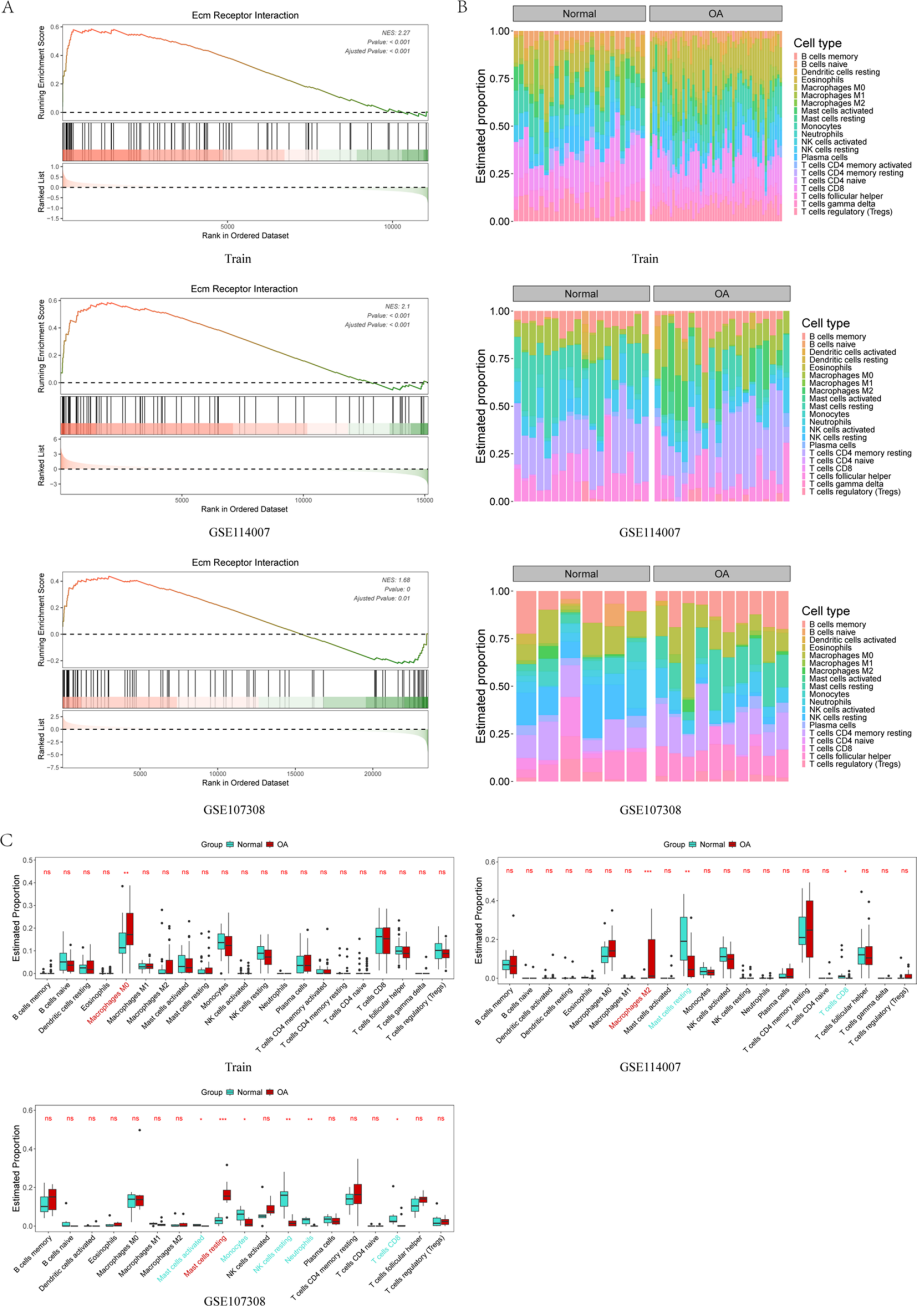
(**A**) GSEA analysis results for the training and validation sets, showing that the cytokine-cytokine receptor interaction pathway is significantly activated in all datasets. (**B**) Immune infiltration analysis results for the training and validation sets, highlighting the percentage of 22 immune cell types in normal and OA groups. (**C**) Furthermore, it shows the differences in immune cell infiltration between the normal and OA groups, with the normal group marked in green and the OA group in red. No common immune infiltration trends were observed among the datasets

GO enrichment analysis revealed OA changes in biological process (BP), cellular component (CC), and molecular function (MF) (Fig. 3A). We found that the biological process was significantly enriched in neuron projection regeneration in both training set and validation set, and the molecular function was significantly enriched in extracellular matrix binding and integrin binding. However, in terms of cellular components, the training and validation sets did not have a common intersection entry. KEGG enrichment analysis results showed that these differentially expressed genes were significantly enriched in PI3K-Akt signaling pathway (Fig. 3B). However, in the following GSEA analysis, both in the training set and the validation set, No activation of PI3K-Akt signaling pathway was found in either training or validation sets. Instead, the ECM_receptor_interaction pathway was significantly activated in all data sets (Fig. 4A). These results suggest that ECM_receptor_interaction pathway may play a key role in OA development.

### Immune infiltration analysis did not find significant difference between OA and normal subjects

To explore the significant difference of immune cell infiltration in cartilage between OA patients and healthy controls, we performed a comprehensive immune infiltration analysis. The bar chart (Fig. 4B) shows the percentages of 22 immune cells in the normal group and the OA group, while the violin chart shows the specific differences of these 22 immune cells between the two groups in detail (Fig. 4C). The analysis results showed that M0 macrophages were significantly higher in the OA group than in the normal group in the training set. In GSE114007, the OA group showed a significant increase in M2 macrophages, while in GSE107308, the level of resting mast cells was significantly increased in the OA group. However, our analysis did not identify any specific immune cells that showed a significant increase or decrease trend in both the training and validation sets.

### Three core genes were selected by the 113 combined machine learning algorithm

**Fig. 5:**
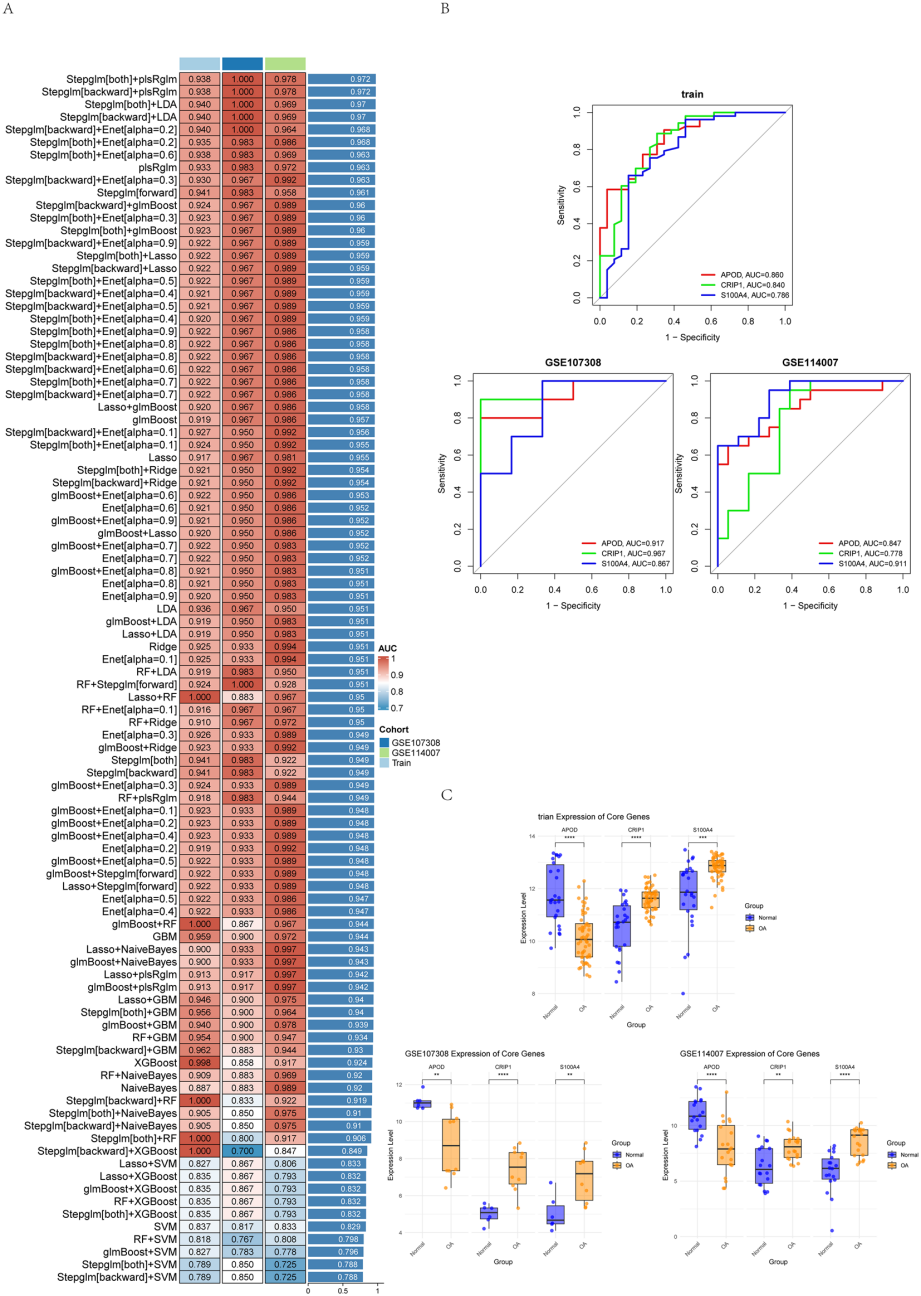
(**A**) In-depth analysis of 113 machine learning algorithms to identify core genes across all datasets, with the plsRglm+Stepglm model producing the highest average AUC value. (**B**) The plsRglm+Stepglm algorithm identified three core genes and generated ROC curves for these genes in the training and validation sets, with AUC values exceeding 0.75 across all datasets. (**C**) Expression levels of the three core genes in normal and OA groups across different datasets, showing that in all datasets, APOD is significantly downregulated in OA patients, while CRIP1 and S100A4 are significantly upregulated

Based on 113 combined machine learning algorithms, 7 differential genes were further screened to identify the most critical core genes. In both the training set and the validation set, multiple models showed excellent performance, with AUC values exceeding 0.90, which showed the significant predictive power of the different combinations of algorithms (Fig. 5A). In particular, the ROC curve drawn by the plsRglm + Stepglm model showed the best prediction effect on the training and validation sets, and its average AUC value was the highest among all the models. Three core genes were selected by this model. Subsequently, we plotted the ROC curves of these three core genes and found that their AUC values were all more than 0.75 (Fig. 5B), indicating that these genes had a good ability to distinguish the normal group from the OA group. In addition, the bar graph of gene expression showed that APOD was significantly decreased in OA patients compared with normal population, while CRIP1 and S100A4 were significantly increased in OA patients (Fig. 5C). The combined ROC curve and gene expression analysis results showed that APOD, CRIP1 and S100A4 could significantly distinguish the normal group from the OA group. Therefore, we confirmed APOD, CRIP1 and S100A4 as core genes.

### qR-PCR was used to verify the actual expression of core genes

**Fig. 6:**
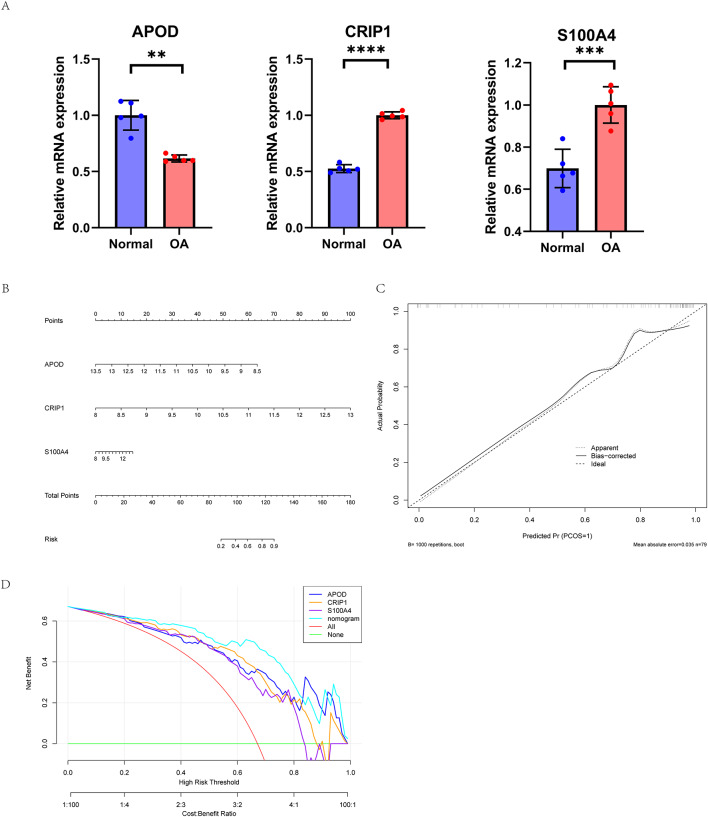
(**A**) PCR experiments further confirm the significant downregulation of APOD and upregulation of CRIP1 and S100A4 in OA model chondrocytes. (**B**) Diagnostic model based on APOD, CRIP1, and S100A4; (**C**) Nomogram calibration curve analysis indicates high consistency between predicted and actual disease incidence rates, further validating the accuracy of the diagnostic model; (**D**) DCA curve of the nomogram prediction, showing significant clinical net benefit

In order to verify the expression of core genes selected by bioinformatics analysis in real samples, we performed quantitative real-time PCR experiments for these three core genes, and each set of experiments was repeated five times. The results showed that APOD was significantly decreased and CRIP1 and S100A4 were significantly increased in the OA group compared with the normal group (Fig. 6A). The expression trend of PCR results was highly consistent with the results of public database analysis.

### Construction of OA diagnostic model

Based on the above methods, we successfully constructed a diagnostic model for OA. Nomogram indicates APOD.

The and CRIP1 genes occupy a major role in the diagnosis, whereas the S100A4 gene is of lesser importance (Fig. 6B). To evaluate the calibration of the prediction model, calibration curves were also drawn. In Fig. 6C, the abscisce is the predicted event rate, and the ordinate is the observed actual event rate. We can see that the predicted curve and the ideal calibration curve are relatively close in most ranges, indicating that the model has a good calibration degree, suggesting that the prediction model has a high accuracy. In addition, clinical decision curve analysis showed that the three-gene combination diagnostic model was above the “net benefit of treatment for all” and “net benefit of no treatment for all” lines in all intervals, that is, all threshold probability ranges, indicating that the model had a significant net clinical benefit in predicting OA at all times (Fig. 6D).

## Discussion

In this study, a comprehensive transcriptome analysis of osteoarthritis (OA) cartilage tissue was performed, which was explored in depth based on the results of six datasets. GO, KEGG and GSEA analyses showed that the activities of extracellular matrix binding, integrin binding and extracellular matrix receptor interaction pathways were closely related to the occurrence and development of OA. In addition, the results of immune infiltration analysis showed that different sequencing technologies in the chip dataset and the high-throughput dataset revealed different immune cell infiltration status. Using 113 machine learning algorithms, we identified three core genes (APOD, CRIP1 and S100A4), which were further validated by PCR. The final diagnostic model showed that these three core genes had good diagnostic efficacy in distinguishing normal controls from OA patients. Therefore, we identified these three core genes as important biomarkers for chondrocyte function in OA.

GSEA analysis helped us to identify the key signaling pathways that were activated or inhibited during OA progression. Although previous studies have mainly focused on the significant pathway changes in OA synovial tissue, the OA cartilage tissue is relatively scarce [18–20]. It has been reported that some inflammation-related pathways such as neutrophil extracellular traps, cell adhesion molecule pathway, cytokine receptor interaction and systemic lupus erythematosus are significantly activated in OA [21, 22]. At the same time, a number of studies have confirmed the significant up-regulation of pathways related to extracellular matrix [23]. The unique finding of this study is that the ECM_RECEPTOR_INTERACTION pathway was significantly activated in both microarray and high-throughput datasets, revealing its possible key role in the involvement of OA chondrocytes in the pathological process.

Previous studies have simultaneously analyzed synovial and cartilage tissues for immune infiltration [24]. However, the main cellular components of synovial tissue and cartilage tissue are different, and Smith believes that the main cells of synovial tissue are “type A synoviocytes” and “type B synoviocytes“ [25, 26]. Cartilage, on the other hand, is made up of a single cell type, chondrocytes, so using data sets from different tissues must theoretically be quite different [27, 28]. In this study, for the sake of rigor, we performed immune infiltration analysis on a single component (cartilage tissue) using both microarray data sets and high-throughput data sets, and the results were quite different. Our study highlights the limitations of inferencing immune infiltration based on existing immune cell gene expression matrices, calling for the need to combine specific experimental data to verify the true proportion of immune cells in tissues.

For the core gene APOD, the results of our study are not only consistent with the results of database analysis, but also with those of previous studies [29–31]. Multiple studies have shown that compared with the normal group. The expression level of APOD was lower in OA group. Zhang Gang’s study demonstrated that APOD can inhibit IL-1β-induced chondrocyte inflammation, degeneration and apoptosis, and it plays a protective role in the progression of OA through the PI3K/AKT/mTOR signaling pathway [32]. Xu Wenbo’s study also demonstrated that APOD could promote the proliferation of chondrocytes, reduce the apoptosis of chondrocytes, reduce oxidative stress, and delay the progression of OA. The conclusions of the two scholars coincide [33].

Proteomic analysis of synovial fluid from knee joints with anterior cruciate ligament (ACL) injury found that CRIP1 protein was significantly increased after ACL injury. However, this study was based on synovial fluid rather than cartilage [34]. Consistent with the trend of bioinformatics analysis in this study, the mRNA expression level of CRIP1 was significantly increased in the OA synovial tissue compared with the normal group (*p* < 0.001) [35]. However, the CRIP1 gene has not been previously studied in OA cartilage in vitro. The results of in vitro experiments -PCR showed that CRIP1 was highly expressed in OA chondrocytes, which is very important. Previous studies have shown that high expression of CRIP1 is associated with poor clinical prognosis in a variety of cancers, including multiple myeloma, hepatocellular carcinoma, gastric cancer, cervical cancer, and colorectal cancer [36–40]. CRIP1 gene affects the proliferation, apoptosis, death, migration and other outcomes of tumor cells through different mechanisms in different cancers. Therefore, we believe that CRIP1 gene can continue to explore its specific mechanism in the occurrence and development of OA.

Both DE proteomics and Western blot showed that S100A4 expression in synovial fibroblasts of OA patients was significantly higher than that of healthy people [41]. Early microarray and real-time PCR results showed that S100A4 was significantly upregulated in chondrocytes of OA patients. Moreover, Western blot and immunohistochemistry also confirmed that S100A4 expression was increased compared with that of normal people. In addition, Raghunatha R Yammani studied the specific mechanism by which IL-7 stimulated the secretion of S100A4 by chondrocytes by activating the JAK/STAT signaling pathway [42–44]. The results of PCR in this study were consistent with the trend of previous studies, which further proved the importance of S100A4 in the development of OA.

In summary, this study identified three important biomarkers and significantly activated ECM receptor interaction pathways in OA chondrocytes involved in disease development, and established an effective diagnostic model based on the three core genes. The greatest value of this study is to highlight the importance of the ECM receptor interaction pathway and CRIP1, which have not attracted the attention of other researchers until now. However, the limitation of this study is that although the up-regulation of CRIP1 in OA has been preliminarily proved by PCR experiments, WB, immunohistochemistry and animal experiments have not been performed to analyze the specific mechanism. Future research should focus on the functional experiments of CRIP1, and we also expect that future research can make more profound progress in this field, so as to lay the foundation for exploring the changes of chondrocytes regulating OA at the gene level.

## Conclusion

In this study, a comprehensive transcriptome analysis of osteoarthritis (OA) cartilage tissue was performed, which revealed the key role of extracellular matrix receptor interaction pathway activation in the occurrence and development of OA. In addition, the results showed that the significant down-regulation of APOD and the significant up-regulation of CRIP1 and S100A4 in cartilage tissue may be important factors in the regulation of OA progression. Especially, CRIP1 has not been fully investigated in OA cartilage tissue in vitro and in vivo. These findings not only deepen our understanding of the pathological mechanism of OA, but also provide a new perspective for future research.

## Data Availability

The datasets used in this study can be found in the Gene Expression Omnibus (GEO) at the following link: https://www.ncbi.nlm.nih.gov/geo/.

## Abbreviations

AUC: Area under the curve
BP: Biological process
CC: Cell composition
DEGs: Differentially expressed genes
GEO: Gene expression omnibus
GSEA: Gene set enrichment analysis
GO: Gene ontology
KEGG: Kyoto encyclopedia of genes and genomes
MF: Molecular function
NES: Normalized enrichment score
ROC: Receiver operating characteristic
OA: Osteoarthritis
